# CYTL1 regulates bone homeostasis in mice by modulating osteogenesis of mesenchymal stem cells and osteoclastogenesis of bone marrow-derived macrophages

**DOI:** 10.1038/s41419-018-1284-4

**Published:** 2019-01-18

**Authors:** Youngnim Shin, Yoonkyung Won, Jeong-In Yang, Jang-Soo Chun

**Affiliations:** National Creative Research Initiatives Center for Osteoarthritis Pathogenesis and School of Life Sciences, Gwangju Institute of Science and Technology, Gwangju, 61005 Korea

## Abstract

We previously showed that mice with knockout of *Cytl1*, a functionally uncharacterized cytokine candidate, exhibit normal endochondral ossification and long-bone development. Here, we investigated the potential functions of CYTL1 in bone homeostasis. We found that *Cytl1*^*−/−*^ mice exhibited higher bone mass than wild-type littermates and resisted ovariectomy-induced bone resorption. This led us to investigate the functions of CYTL1 in the osteogenesis and osteoclastogenesis of bone marrow-derived stem cells. CYTL1 was down-regulated during the osteogenesis of human mesenchymal stem cells (hMSCs). The osteogenesis of hMSCs was inhibited by overexpression or exogenous treatment of CYTL1, but enhanced by CYTL1 knockdown. CYTL1 decreased osteogenesis by inhibiting RUNX2 and promoted proliferation among undifferentiated hMSCs, but stimulated apoptosis among osteogenically differentiating cells. Finally, *Cytl1*^*−/−*^ mice exhibited inhibition of osteoclast activity and the osteoclastogenesis of bone marrow-derived macrophages. Our results collectively suggest that CYTL1 negatively regulates the osteogenesis of MSCs and positively regulates osteoclastogenesis to modulate bone mass in mice.

## Introduction

Cytokine-like 1 (CYTL1) was originally cloned from CD34^+^ human bone marrow and cord blood as a secreted cytokine candidate^1^. Although the detailed functions of CYTL1 remain largely unknown, it exhibits structural similarities with the chemokine, CCL2, and the CCL2 receptor (CCR2) has been suggested as a potential receptor of CYTL1^2^. Recent studies revealed that CYTL1 exerts diverse biological functions in various model animals. To date, CYTL1 has been associated with the growth and metastasis of neuroblastoma cells^3^, embryonic implantation (as an ovarian-hormone-dependent protein)^4^, the chemoattraction of monocytes and macrophages^5^, pars tuberalis morphogenesis^6^, cardiac fibrosis^7^, etc.

We previously showed that CYTL1 regulates the chondrogenesis of mesenchymal cells as a novel autocrine factor^8^. CYTL1 expression is low in mouse limb bud mesenchymal cells, dramatically increases during their micromass culture-induced chondrogenesis, and thereafter decreases during the hypertrophic maturation of differentiated chondrocytes. The application of exogenous CYTL1 or lentivirus-mediated overexpression of CYTL1 was shown to enhance the chondrogenic differentiation of mouse limb bud mesenchymal cells in micromass culture without affecting the hypertrophic maturation of chondrocytes^9^. However, we subsequently found that deletion of *Cytl1* in mice (*Cytl1*^*−/−*^) does not affect chondrogenesis or cartilage development^9^, suggesting that CYTL1 is not a dominant regulator of these processes. Notably, CYTL1 expression is markedly decreased in the osteoarthritic cartilage of humans and experimental mice, and *Cytl1*^*−/*−^ mice are more sensitive than their wild-type (WT) counterparts to osteoarthritic cartilage destruction^9^. Therefore, CYTL1 appears to be required for the maintenance of cartilage homeostasis, and loss of CYTL1 function seems to be associated with experimental osteoarthritic cartilage destruction in mice.

We also found previously that *Cytl1*^*−/−*^ mice exhibit normal endochondral ossification and long-bone development, as assessed in E16.5 and E18.5 embryos and 2-week-old postnatal mice, with similar body sizes seen in *Cytl1*^*−/*−^ mice and wild-type (WT) littermates^9^. However, the role of CYTL1 in regulating parameters of bone homeostasis, such as bone mass, had not been investigated previously. Bone homeostasis is primarily regulated by the osteogenesis of bone marrow-derived mesenchymal stem cells (BM-MSCs) and the osteoclastogenesis of bone marrow-derived macrophages (BMMs)^10^. BM-MSCs are multipotent stem cells that can be induced to differentiate into multiple mesenchymal lineages, including osteoblasts, adipocytes, and chondrocytes^11^. The differentiation of BM-MSCs is regulated by lineage-specific transcription factors, such as RUNX2 for osteoblasts, C/EBPα and PPARγ for adipocytes, and SOX9 for chondrocytes^12^. Multiple molecules (e.g., growth factors and cytokines) and signaling pathways are known to regulate the activities of these lineage-specific transcription factors during BM-MSC differentiation^13,14^. This differentiation plays important roles in tissue regeneration and remodeling, as bone marrow-derived osteoblasts and chondrocytes regulate the modeling/remodeling of bone and cartilage, respectively^15,16^. In contrast, osteoclasts, which are primarily responsible for bone resorption, differentiate from BMMs via a process that critically involves the transcription factor, NFATc1^17^.

Given our previous finding that *Cytl1*^−*/*−^ mice exhibited increased bone mass compared with WT littermates, we set out to investigate whether the modulation of bone mass by CYTL1 is due to alterations in the osteogenesis of MSCs and/or the osteoclastogenesis of BMMs. Here, we report in vitro results indicating that CYTL1 is down-regulated during osteogenic differentiation, and that the osteogenesis of MSCs is inhibited by overexpression of CYTL1 or treatment of exogenous CYTL1 but promoted by knockdown of CYTL1. In contrast to this negative regulation of osteogenesis, we show that CYTL1 positively regulates the osteoclastogenesis of BMMs in vitro. Consistent with these findings, *Cytl1*^−*/−*^ mice exhibit enhanced bone mass and resistance to ovariectomy-induced bone loss. Our results collectively suggest that CYTL1 regulates bone mass by negatively regulating osteogenesis and positively regulating osteoclastogenesis.

## Results

### *Cytl1*^*−/−*^ mice exhibit increased bone mass and resistance to ovariectomy-induced bone resorption

**Fig. 1:**
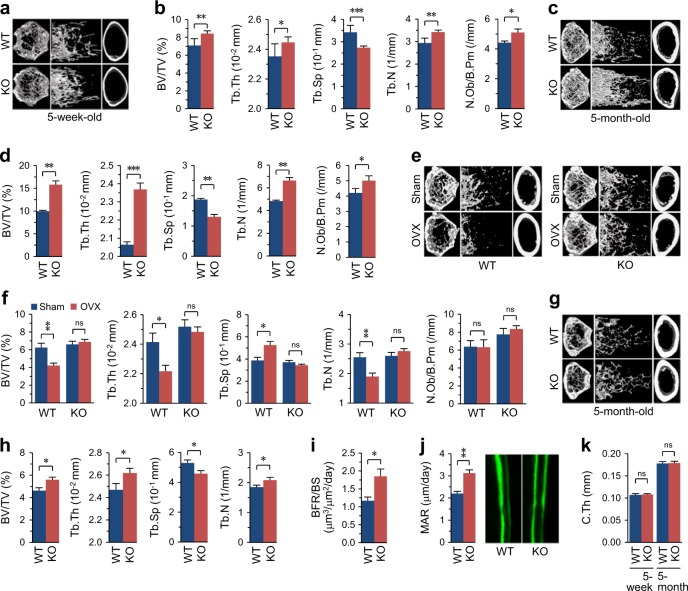
*Cytl1*^−*/−*^ mice exhibit enhanced bone mass and resistance to ovariectomy-induced bone resorption. **a**, **b** Representative 3-dimensional reconstructive images of trabecular and cortical bone from the distal femur metaphyses of 5-week-old WT and *Cytl1*^*−/−*^ (KO) male mice (**a**). The BV/TV (bone volume/total volume), Tb.Th (trabecular thickness), Tb.Sp (trabecular separation), Tb.N (trabecular number), and N.Ob/B.Pm (Number of  osteoblast per bone perimeter) were determined from 10 different mice (**b**). **c**, **d** Representative 3-dimensional reconstructive images of 5-month-old WT and KO male mice (**c**) and BV/TV, Tb.Th, Tb.Sp, Tb.N, and N.Ob/B.Pm from 10 different mice (**d**). **e**, **f** Representative 3-dimensional reconstructive images of trabecular and cortical bone from the distal femur metaphyses obtained 8 weeks after ovariectomy (OVX) or sham operation performed on 10-week-old WT and KO female mice (**e**). The BV/TV, Tb.Th, Tb.Sp, Tb.N, and N.Ob/B.Pm were determined from 8 different mice (**f**). Littermates of WT or KO mice were either sham operated or subjected to OVX. **g**, **h** Representative 3-dimensional reconstructive images of trabecular and cortical bone from the distal femur metaphyses of 5-month-old female KO mice and WT littermates (**g**) and BV/TV, Tb.Th, Tb.Sp, and Tb.N from 7 different mice per group (**h**). **i**, **j** New bone formation was measured by double calcein labeling. Bone formation rate/bone surface (**i**) and the mineral apposition rate (MAR) (**j**) were measured at the distal femur metaphyses of 5-month-old WT and KO mice (n = 8 mice/group). **k** The cortical bone thickness (C.Th) was determined from KO mice and WT littermates (*n* = 10 mice/group). Data represent the means ± SEM from the indicated numbers of mice; *p < 0.05, **p < 0.005, and ***p < 0.0005, as determined by two-tailed *t*-test; ns not significant

We previously showed that CYTL1 regulates cartilage homeostasis without critically affecting cartilage development^8,9^ and further reported that *Cytl1*^−*/*−^ mice exhibit normal endochondral ossification and long-bone development^9^. Here, we first examined whether CYTL1 regulates the homeostasis of long bones by examining bone mass in *Cytl1*^−*/−*^ mice and WT littermates. Micro-computed tomography (μCT) was used to perform 3-dimensional analysis of the metaphyseal femoral regions of the long bones of 5-week-old male WT and *Cytl1*^*−/*−^ mice. Compared with WT littermates, *Cytl1*^*−/*−^ mice exhibited increases in their bone mass, bone volume per trabecular volume (BV/TV), and trabecular thickness (Tb.Th), with a concomitant decrease in their trabecular separation (Tb.Sp) (Fig. 1a, b). The trabecular number (Tb.N) and number of osteoblasts per bone perimeter (N.Ob/B.Pm) in *Cytl1*^−*/*−^ mice was also significantly higher than that in WT littermates (Fig. 1b). Compared with these 5-week-old mice, 5-month-old mice exhibited more significant increases in bone mass, BV/TV, Tb.Th, Tb.N, and N.Ob/B.Pm, with a concomitant decrease in Tb.Sp (Fig. 1c, d). These results indicate that the loss of CYTL1 function results in increased bone mass in mice.

To further elucidate the in vivo significance of CYTL1 in bone homeostasis, we examined whether *Cytl1* knockout in mice could mitigate ovariectomy (OVX)-induced bone resorption. To this end, WT female littermates and KO female littermates were subjected to either sham operation or OVX followed by bone phenotype analysis. As expected, μCT analyses of the distal femurs of ovariectomized WT mice revealed trabecular bone loss (Fig. 1e) with decreased BV/TV, Tb.Th, and Tb.N, increased Tb.Sp, and no change in N.Ob/B.Pm (Fig. 1f). However, compared with sham-operated littermates, the ovariectomized *Cytl1*^*−/−*^ mice did not exhibit any change in the examined bone phenotypes (Fig. 1e, f). Because the sham-operated WT and *Cytl1*^−/−^ female mice were not littermates, we failed to detect significant differences in the μCT bone phenotypes of these groups (Fig. 1e, f). However, analysis μCT and bone phenotypes (BV/TV, Tb.Th, Tb.Sp, and Tb.N) between female *Cytl1*^*−/−*^ mice and WT littermates exhibited significant differences, similar to those observed in male mice (Fig. 1g, h). We additionally found that, compared with WT littermates, 5-month-old male *Cytl1*^*−/−*^ mice exhibited significant increases in the bone formation rate per bone surface (BFR/BS, μm^3^/μm^2^/day) (Fig. 1i) and the mineral apposition rate (MAR; μm/day) (Fig. 1j). In contrast to the above-described phenotypic changes of trabecular bone, our μCT imaging (Fig. 1a, c, e, g) and analysis of cortical bone thickness indicated that there was no marked difference between *Cytl1*^*−/−*^ mice and WT littermates in the context of cortical bone (Fig. 1k).

### Differential expression pattern of CYTL1 during the mesenchymal-lineage differentiation of hMSCs

**Fig. 2:**
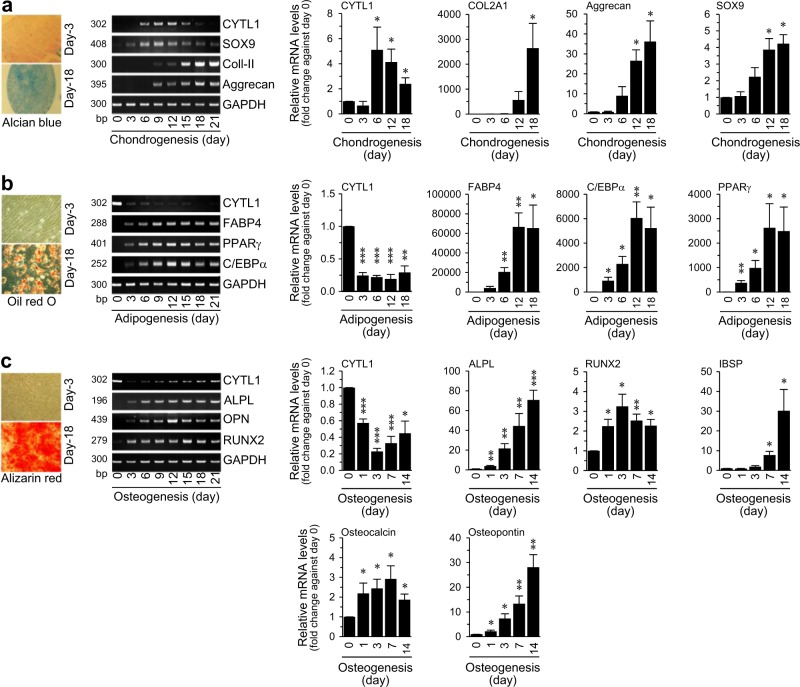
Differential expression of CYTL1 during hMSC differentiation. **a–c** Chondrogenic (**a**), adipogenic (**b**), and osteogenic (**c**) differentiation of hMSCs. Representative lineage-specific staining images are presented in the left panels. Representative RT-PCR images and qRT-PCR analyses (*n* = 8) of CYTL1 and the indicated differentiation markers are presented in the middle and right panels. Data represent the mean ± SEM from six independent experiments. *P*-values were determined by one-way ANOVA

To elucidate the possible mechanisms underlying the CYTL1-mediated regulation of bone mass, we examined the functions of CYTL1 in the osteogenic differentiation of hMSCs. We first examined the mRNA levels of CYTL1 during the forced differentiation of hMSCs into chondrocytes, adipocytes, and osteoblasts. hMSC differentiation was confirmed by lineage-specific staining and detection of lineage-specific markers, as follows: Alcian blue staining and detection of SOX9, type II collagen (Coll-II, COL2A1), and aggrecan were used to identify chondrocytes; Oil red O staining and detection of FABP4 (fatty acid-binding protein 4), C/EBPα (CCAAT-enhancer-binding protein α), and PPARγ (peroxisome proliferator-activated receptor γ) were used to identify adipocytes; and Alizarin red S staining and detection of ALPL (alkaline phosphatase), RUNX2 (Runt-related transcription factor 2), IBSP (integrin binding sialoprotein), osteocalcin, and osteopontin were used to identify osteoblasts^12,14^ (Fig. 2a–c). Our results revealed that CYTL1 expression was transiently increased during the chondrogenesis of hMSCs (Fig. 2a) but decreased during adipogenesis, wherein it remained at a low level until the late phase of differentiation (Fig. 2b). In contrast, CYTL1 expression was markedly decreased at the early phase (day 3) of osteogenesis and exhibited a slight recovery during the late phase of differentiation (Fig. 2c).

### Overexpression of CYTL1 inhibits the osteogenesis of hMSCs without affecting their adipogenesis or chondrogenesis

**Fig. 3:**
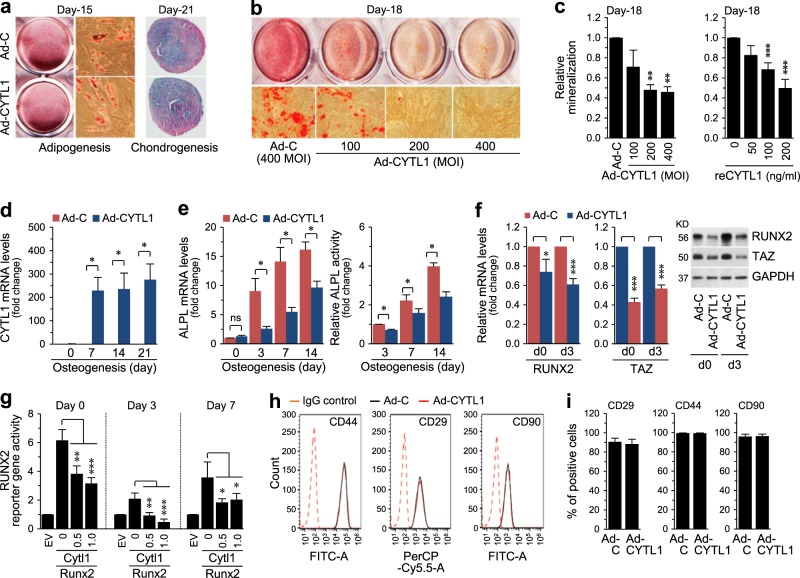
Overexpression of CYTL1 inhibits the osteogenesis of hMSCs. **a** Representative staining images of the adipogenic and chondrogenic differentiations of hMSCs (*n* = 5). **b** Representative staining images of the osteogenic differentiation of hMSCs infected with Ad-C or Ad-CYTL1 (*n* = 10). **c** Quantitation of Alizarin red S staining in hMSCs infected with Ad-C or Ad-CYTL1 (left, *n* = 10) or treated with recombinant CYTL1 protein (right, *n* = 8). **d**, **e** The CYTL1 mRNA level (**d**, *n* = 7), ALPL enzyme activity, and ALPL mRNA level (**e**, *n* = 5) in hMSCs (day 0) and osteogenically differentiating hMSCs infected with Ad-C or Ad-CYTL1. **f** mRNA and protein levels of RUNX2 and TAZ in hMSCs (day 0) or osteogenically differentiating hMSCs (day 3) infected with 400 MOI of Ad-C or Ad-CYTL1 (*n* = 6). **g** RUNX2 reporter gene activity in hMSCs (day 0) or osteogenically differentiating hMSCs on days 3 or 7 after transfection with empty vector (EV, 1 μg) or expression vectors encoding mouse *Runx2* (1 μg) or mouse *Cytl1* (amounts indicated, μg) (*n* = 5). **h**, **i** Expression of hMSC markers (CD44, CD29, and CD90) in hMSCs infected with 400 MOI of Ad-C or Ad-CYTL1 (*n* = 4). Data represent the means ± SEM of the indicated number of independent experiments; *p < 0.05, **p < 0.005, and ***p < 0.0005, as determined by one-way ANOVA (**c, g**) or two-tailed *t*-test (**d**, **e**, **f**, **i**)

Because the differential expression pattern of CYTL1 suggested that it might play distinct functions during the tri-lineage differentiation of hMSCs, we examined the functions of CYTL1 in hMSC differentiation via gain-of-function (overexpression or exogenous CYTL1) and loss-of-function (knockdown) approaches. We overexpressed CYTL1 in hMSCs by infecting cells with an adenovirus encoding human CYTL1 (Ad-CYTL1), and induced the cells to undergo tri-lineage differentiation. Overexpression of CYTL1 did not affect chondrogenesis or adipogenesis, as determined by Alcian blue or Oil red O staining, respectively (Fig. 3a). However, compared to infection of hMSCs with control empty adenovirus (Ad-C), infection of Ad-CYTL1 dose-dependently inhibited the osteogenesis of hMSCs, as determined by Alizarin red S staining at culture day 18 (Fig. 3b). Quantitation of mineralization at culture day 18 also indicated that Ad-CYTL1 or recombinant CYTL1 (reCYTL1) dose-dependently inhibited osteogenesis (Fig. 3c). Overexpression of CYTL1 at various days of osteogenic differentiation in hMSCs (Fig. 3d) reduced the expression and activity of alkaline phosphatase (ALPL) (Fig. 3e). Consistent with this finding, overexpression of CYTL1 in undifferentiated hMSCs (day 0) and osteogenically differentiating cells (day 3) reduced the mRNA and protein levels of RUNX2, which is an upstream regulator of ALPL^12^ (Fig. 3f). The mRNA and protein levels of TAZ, a co-activator of RUNX2^18^, were also decreased in CYTL1-overexpressing and osteogenically differentiating hMSCs (Fig. 3f). Furthermore, CYTL1 overexpression significantly inhibited RUNX2 reporter gene activity in undifferentiated hMSCs and in osteogenically differentiating hMSCs on days 3 or 7 of induction (Fig. 3g). When we further examined whether CYTL1 modulates MSC phenotypes, our FACS (fluorescence-activated cell sorting) analysis revealed that overexpression of CYTL1 did not affect the expression of the MSC markers, CD29, CD44, and CD90 (Fig. 3h, i). Taken together, our results indicate that CYTL1 negatively regulates the osteogenesis of hMSCs by inhibiting RUNX2, but this does not affect hMSC phenotypes.

### Knockdown of CYTL1 stimulates the osteogenesis of hMSCs

**Fig. 4:**
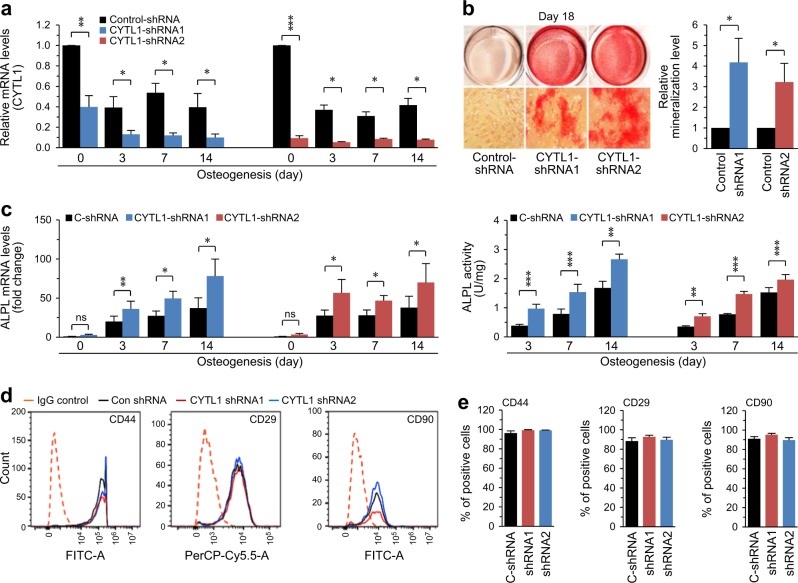
Knockdown of CYTL1 promotes osteogenesis of hMSCs. **a–c** hMSCs were transfected with lentiviruses (25 MOI) expressing control shRNA or two different CYTL1-targeting shRNAs and cultured under the osteogenic differentiation-inducing conditions for the indicated number of days. **a** CYTL1 mRNA levels *(n* = 6). **b** Representative images of Alizarin red S staining and relative mineralization levels (*n* = 7). **c** ALPL mRNA level and enzyme activity (*n* = 7). **d, e** Expression of hMSC markers (CD44, CD29, and CD90) in hMSCs infected with lentiviruses (20 MOI) expressing control shRNA or two different CYTL1-targeting shRNAs (**d**, *n* = 5). Data represent the means ± SEM of the indicated number of independent experiments (**e**); *p < 0.05, **p < 0.005, and ***p < 0.0005, as determined by two-tailed *t*-test; ns not significant. See also Figure S1

We next examined the effects of CYTL1 knockdown on the osteogenesis of hMSCs. To knock down CYTL1, we infected hMSCs with lentiviruses carrying shRNAs specific to human CYTL1 (Supplementary Table S1). Compared with the control shRNA, the two different tested CYTL1 shRNAs (CYTL1-shRNA1 and CYTL1-shRNA2) both significantly down-regulated the mRNA levels of CYTL1 in undifferentiated hMSCs and in osteogenically differentiating cells at days 3, 7, and 14 of induction (Fig. 4a). Knockdown of CYTL1 significantly promoted osteogenesis, as determined by Alizarin red S staining and quantitation of mineralization at culture day 18 (Fig. 4b). Knockdown of CYTL1 at various days of osteogenic differentiation in hMSCs was found to significantly enhance the expression and activity of ALPL (Fig. 4c). Similar to these effects of shRNA-mediated CYTL1 knockdown, that mediated using a specific siRNA (Supplementary Table S1) also promoted the osteogenesis of hMSCs (Supplementary Fig. S1). Knockdown of CYTL1 did not affect the expression levels of the MSC markers, CD29, CD44, and CD90 (Fig. 4d, e), further supporting our proposal that CYTL1 negatively regulates the osteogenesis of hMSCs without affecting their basal MSC phenotypes.

### CYTL1 promotes the proliferation of undifferentiated hMSCs and induces apoptosis of osteogenic-differentiating hMSCs

**Fig. 5:**
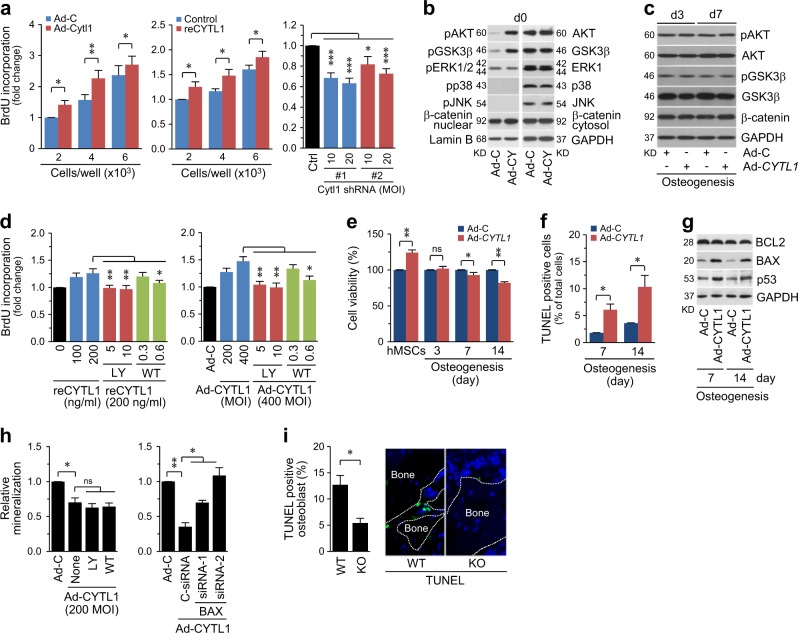
Overexpression of CYTL1 promotes the proliferation of undifferentiated hMSCs and induces apoptosis of osteogenically differentiating hMSCs. **a** hMSCs were infected with 400 MOI of Ad-C or Ad-CYTL1, treated with 200 ng/ml of recombinant CYTL1 (reCYTL1), or infected with 10 or 20 MOI of lentivirus-based control or CYTL1 shRNAs. hMSC proliferation was determined by BrdU incorporation assay (adenovirus; *n* = 8, shRNA; *n* = 4). **b**, **c** hMSCs were infected with 400 MOI of Ad-C or Ad-CYTL1 and cultured for 0 (**b**), 3, and 7 days under osteogenic differentiation-inducing conditions (**c**). The indicated proteins were detected by western blotting. **d** hMSCs were treated with reCYTL1 (left) or infected with 400 MOI of Ad-C or Ad-CYTL1 (right) in the absence or presence of the AKT inhibitors, LY294002 (5, 10 μM) or Wortmannin (0.3, 0.6 μM). Proliferation of hMSCs was determined by BrdU incorporation assay (*n* = 8). **e**–**h** hMSCs were maintained under non-differentiating or osteogenic-differentiation-inducing conditions for the indicated number of days. Cell viability was determined by the MTS assay (**e**, *n* = 7). Apoptotic cells were quantified by TUNEL staining (**f**, *n* = 5). Western blotting was performed to detect BCL2, BAX, and p53; representative images are shown (**g**, *n* = 5). **h** Quantitation of Alizarin red S staining in hMSCs infected with 200 MOI of Ad-C or Ad-CYTL1 in the absence or presence of the AKT inhibitors, LY294002 (10 μM) or Wortmannin (0.6 μM) (left, *n* *=* 14) and cells transfected with 10 nM of control siRNA or two different BAX siRNA (right, *n* = 4). **i** Representative TUNEL staining image and counting of apoptotic osteoblasts from KO mice and WT littermates (*n* = 10 mice per group). Data represent the means ± SEM of the indicated number of independent experiments; *p < 0.05, **p < 0.005, and ***p < 0.0005, as determined by two-tailed *t*-test; ns not significant

To elucidate the mechanisms underlying the ability of CYTL1 to negatively regulate osteogenesis, we examined the contributions of CYTL1 to the proliferation of undifferentiated hMSCs, which increases the pool of osteoprogenitor cells. BrdU incorporation assays revealed that the Ad-CYTL1-mediated overexpression of CYTL1 or treatment of recombinant CYTL1 (reCYTL1) in hMSCs significantly promoted proliferation, whereas shRNA-mediated knockdown of CYTL1 suppressed hMSC proliferation (Fig. 5a). Consistent with this ability to promote hMSC proliferation, CYTL1 overexpression increased the activating phosphorylations of AKT and GSK3β (Fig. 5b), which function in a signaling pathway that regulates cell survival and proliferation^19^. Indeed, inhibition of AKT with LY294002 or Wortmannin dose-dependently abolished the stimulatory effects of CYTL1 overexpression or reCYTL1 treatment on the proliferation of undifferentiated hMSCs (Fig. 5d).

In contrast to these effects in undifferentiated hMSCs, the overexpression of CYTL1 in osteogenically differentiating hMSCs (days 3 and 7) did not affect the phosphorylation of AKT or GSK3β (Fig. 5c). However, MTS [3-(4,5-dimethylthiazol-2-yl)-5-(3-carboxymethoxyphenyl)-2-(4-sulfophenyl)-2H-tetrazolium] assays revealed that cell viability was significantly reduced in CYTL1-overexpressing osteogenically differentiating hMSCs on days 7 and 14 (Fig. 5e). Moreover, the population of TUNEL-positive apoptotic cells was significantly increased by the overexpression of CYTL1 in osteogenically differentiating hMSCs on days 7 and 14 (Fig. 5f). CYTL1 overexpression increased the protein level of BAX without affecting that of BCL2 (Fig. 5g); given that the ratio of BAX to BCL2 determines the susceptibility to apoptosis^20^, this increase in the BAX protein level suggest that the decreased cell viability in CYTL1-overexpressing osteogenically differentiating hMSCs was due to increased apoptotic cell death. Additionally, CYTL1 overexpression in osteogenically differentiating hMSC markedly increased the levels of p53 (Fig. 5g), indicating that p53 regulates BAX up-regulation in this context.

The links between osteogenesis and the ability of CYTL1 to regulate MSC proliferation and osteoblast apoptosis were evaluated by examining the effects of AKT inhibition (with LY294002 or Wortmannin) or BAX knockdown (with specific siRNA) on the osteogenesis of hMSCs. We found that AKT inhibition did not affect the Ad-CYTL1-induced inhibition of osteogenesis, as determined by Alizarin red staining (Fig. 5h). This indicates that the AKT signaling does not directly regulate the osteogenesis of hMSCs, although it increases the pool of osteoprogenitor cells by promoting cell proliferation. These results are consistent with our observation that CYTL1 overexpression in osteogenically differentiating hMSCs does not affect AKT signaling (Fig. 5c). Conversely, knockdown of BAX significantly blocked the Ad-CYTL1-induced inhibition of osteogenesis (Fig. 5h). This clearly indicates that the CYTL1 overexpression-induced up-regulation of BAX (Fig. 5g) is necessary for the ability of CYTL1 to regulate the osteogenesis of hMSCs. Consistently, we found that the numbers of TUNEL-positive apoptotic osteoblasts in trabecular bone were significantly decreased in *Cytl1*^*−/*−^ mice compared with WT littermates (Fig. 5i). Our results collectively support the idea that the CYTL1-mediated regulation of osteoblast apoptosis plays an important role in modulating the bone mass of mice.

### CYTL1 positively regulates osteoclastogenesis in mice

**Fig. 6:**
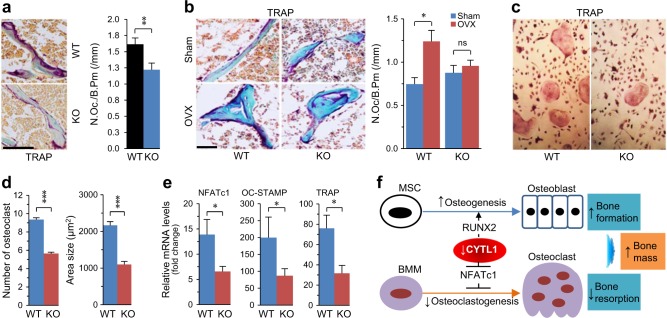
CYTL1 regulates osteoclastogenesis. **a** Representative TRAP-stained images obtained from the metaphyseal regions of the distal femurs of 5-week-old male WT and *Cytl1*^−*/−*^ mice (left panel). The number of osteoclast per bone perimeter (N.Oc./B.Pm) was measured from eight mice per group (right panel). **b** Representative TRAP-stained images obtained from the metaphyseal regions of distal femurs obtained 8 weeks after ovariectomy (OVX) or sham operation of 10-week-old female WT and *Cytl1*^−*/−*^ mice. N.Oc./B.Pm was measured from five mice per group. **c**, **d** BMMs isolated from *Cytl1*^−*/−*^ and WT mice were differentiated into osteoclasts. TRAP staining was performed to detect osteoclasts (**c**), and the numbers and sizes of osteoclasts in each well were quantified (**d**). **e** mRNA levels of the osteoclast marker genes, TRAP, OC-STAMP, and NFATc1, during the osteoclastic differentiation (day 2) of BMMs from *Cytl1*^−*/−*^ and WT mice (*n* = 6). **f** Working model for the mechanism through which CYTL1 regulates bone mass in *Cytl1*^−*/−*^ mice. Data represent the means ± SEM; *p < 0.05, and **p < 0.001, as determined by two-tailed *t*-test; ns not significant. Scale bar = 50 μm

Given that bone mass is determined by the balance between osteoblast-mediated bone formation and osteoclast-mediated bone resorption^10^, we examined whether CYTL1 affects osteoclast functions. We used TRAP (tartrate-resistant acid phosphatase) staining to detect osteoclasts in mice, and quantified the number of osteoclasts per bone perimeter (N.Oc/B.Pm). We found that TRAP staining and N.Oc/B.Pm were significantly reduced in 5-week-old male *Cytl1*^*−/−*^ mice compared with WT littermates (Fig. 6a). Additionally, we observed marked increases of TRAP staining and N.Oc/B.Pm in ovariectomized WT female mice compared to sham-operated WT controls, but no such change was seen in *Cytl1*^*−/−*^ female mice (Fig. 6b). Lastly, we examined whether CYTL1 directly regulates the osteoclastogenesis of BMMs. Indeed, the osteoclastogenesis of BMMs from *Cytl1*^*−/−*^ mice was significantly reduced compared to that of BMMs isolated from WT littermates (Fig. 6c, d). Consistent with this finding, the expression levels of NFATc1, a transcription factor that acts as a key regulator of osteoclastogenesis^17^, and its downstream targets (OC-STAMP and TRAP) were significantly reduced in differentiated BMMs from *Cytl1*^−*/−*^ mice compared with those from WT littermates (Fig. 6e). These results collectively suggest that both increased osteogenesis/osteoblast activity and decreased osteoclastogenesis/osteoclast activity contribute to the increased bone mass seen in *Cytl1*^*−/−*^ mice.

## Discussion

We previously reported that *Cytl1* knockout in mice did not affect endochondral ossification or long-bone development, as examined in E16.5 and E18.5 embryos or 2-week-old postnatal mice^9^. However, we herein demonstrate that *Cytl1*^*−/−*^ mice exhibit enhanced bone mass and resistance to OVX-induced bone resorption. We also demonstrate that the bone mass increase seen in *Cytl1*^−*/*−^ mice is due to decreased osteoclastogenesis of BMMs and increased osteogenesis of MSCs (Fig. 6f). Our results additionally reveal that CYTL1 inhibits the osteogenic differentiation of hMSCs by blocking RUNX2, and that it promotes the proliferation of undifferentiated hMSCs and stimulates the apoptosis of osteogenically differentiating hMSCs by enhancing BAX expression.

The differentiation of MSCs into osteoblasts is pivotal to the processes of bone remodeling^21^. The transcription factor, RUNX2, is a key regulator of osteogenesis in BM-MSCs^12^. RUNX2 promotes the expression of osteogenesis-related genes, including those encoding ALPL, integrin bone sialoprotein (IBSP), and osteocalcin (BGLAP), which are critical regulators of osteoblast differentiation and function^22^. RUNX2-regulated ALPL is directly responsible for the mineralization of the extracellular matrix^23^. ALPL is a membrane-bound enzyme that hydrolyzes inorganic pyrophosphates (PPi) into phosphates (Pi) to form hydroxyapatite, which is required for the initiation of mineralization. Although we did not elucidate how CYTL1 inhibits the expression and activity of RUNX2 and its co-activator, TAZ, our results suggest that the capacity of CYTL1 to inhibit RUNX2 is responsible for the inhibition of ALPL expression/activity that is seen during the osteogenic differentiation of hMSCs and is ultimately responsible for inhibiting mineralization.

We herein demonstrate that CYTL1 promotes the proliferation of undifferentiated hMSCs and stimulates apoptosis among osteogenic hMSCs. Moreover, we show that the CYTL1-mediated regulation of MSC proliferation is not directly associated with its ability to regulate bone mass, although it does function indirectly by increasing the pool of osteoprogenitor cells. In contrast, our results clearly indicate that the BAX-mediated apoptosis of osteoblasts is directly associated with the CYTL1-mediated regulation of bone mass. We show that CYTL1 promotes hMSC proliferation through AKT/GSK3β signaling via a β-catenin-independent pathway. Similarly, a previous report found that GSK-3β promotes the proliferation of megakaryocytic cells through a β-catenin-independent pathway^24^. Although AKT principally functions in cell survival, accumulating evidence indicates that it also regulates mitogenic pathways^25,26^. Additionally, CYTL1 has been reported to show proliferative effects in other cells types, including neuroblastoma cells and endometrial cancer cell lines^3,4^. During osteogenic differentiation, MSCs must first proliferate to increase the population of osteoprogenitors; thereafter, they condense, stop proliferating, and differentiate into osteoblasts. RUNX2, which promotes osteogenic differentiation, exerts an anti-proliferative effect by regulating cell cycle genes^27,28^. Knockdown of RUNX2 up-regulates cyclins A1, B, and E, down-regulates p21, and enhances proliferation among human MSCs, MC3T3 preosteoblasts, and C2C12 mesenchymal cells^29,30^. Therefore, the CYTL1-mediated inhibition of RUNX2 activity had previously appeared to be related to the enhanced proliferation of hMSCs. In contrast, we herein report that CYTL1 stimulates apoptosis in osteogenic-differentiating hMSCs. Thus, our results suggest that CYTL1 exhibits differential effects depending on the type of differentiation. A number of other regulatory factors have been shown to exhibit differentiation-stage-specific functions. For instance, in primary mouse calvarial cells and a murine osteoblast cell line, fibroblast growth factor 1 (FGF1) was found to enhance the proliferation of non-differentiating cells while enhancing the apoptosis of osteogenically differentiating cells^31^.

Although CYTL1 expression is down-regulated during the adipogenic differentiation of hMSCs and transiently up-regulated during the chondrogenic differentiation of these cells, CYTL1 overexpression did not modulate the differentiation of hMSCs into either of these lineages. The expression pattern of CYTL1 during micromass culture-induced chondrogenesis of mouse limb bud mesenchymal cells^8^ was similar to that observed during the chondrogenesis of hMSCs in the present work: in both cases, CYTL1 expression was transiently increased during chondrogenic differentiation. We previously showed that exogenous CYTL1 treatment or lentivirus-mediated overexpression of CYTL1 in mouse limb bud mesenchymal cells enhanced chondrogenic differentiation during micromass culture^8^. In contrast, our current study indicates that the overexpression of CYTL1 in hMSCs did not affect their chondrogenic differentiation. Thus, CYTL1 appears to play different roles in limb bud mesenchymal cells and hMSCs. Although CYTL1 does not appear to be an essential regulator of cartilage development, as *Cytl1*^*−/*−^ mice exhibit normal cartilage development, we previously showed that CYTL1 appears to be required for the maintenance of cartilage homeostasis, and that loss of CYTL1 function seems to be associated with experimental osteoarthritic cartilage destruction in mice^9^.

In the present study, we found that *Cytl1*^*−/−*^ mice exhibit increased bone mass, which is well consistent with the ability of CYTL1 to negatively regulate the osteogenesis of hMSCs. Moreover, we found that CYTL1 negatively regulates osteogenesis by inhibiting the expression and activity of RUNX2. Consistent with these observations, *Runx2*-deficient mice were previously reported to show a deficiency of osteoblasts and a consequent lack of bone formation^32^. Additionally, genetic ablation of *Akp2*, a non-tissue-specific alkaline phosphatase, in mice resulted in skeletal hypomineralization^33^. This phenotype is consistent with those observed in *Cytl1*^*−/−*^ mice. We found that TRAP staining and N.Oc/B.Pm were reduced in *Cytl1*^*−/*−^ mice compared with WT littermates, and ovariectomized *Cytl1*^*−/*−^ mice exhibited reduced bone resorption, as reflected by decreased TRAP staining and osteoclast numbers. These findings indicate that CYTL1 modulates the differentiation and function of osteoclasts. Indeed, our results clearly demonstrate that the osteoclastogenesis of BMMs isolated from *Cytl1*^*−/−*^ mice is decreased compared with those from WT littermates.

CYTL1 exhibits structural similarities with the chemokine, CCL2, which binds and transfers signals through the chemokine receptor, CCR2^2^. Previously, CYTL1 was shown to exhibit chemotactic activity through CCR2 in monocytes/macrophages in vitro^5^ and induce expression of CCR2 in chondrocytes^34^. Therefore, it is interesting to note that the phenotypes of *Cytl1*^*−/−*^ mice are similar to those of mice deficient for *Ccl2* or *Ccr2*; the former displayed an increase in trabecular bone volume due to the inhibition of osteoclastogenesis, while the latter exhibited increased bone mass and decreases in the number, size, and function of osteoclasts^35–37^. However, our reverse transcription-polymerase chain reaction (RT-PCR) analysis failed to detect CCR2 expression in osteogenically differentiating hMSCs under various conditions (data not shown), suggesting that CCR2 may not mediate the ability of CYTL1 to regulate the osteogenesis of hMSCs. Indeed, we previously reported that CCR2 does not mediate the ability of CYTL1 to regulate cardiac fibrosis^7^. In this prior study, we found that CYTL1 and CCL2 both induce the trans-differentiation of fibroblasts to myofibroblasts, and that pharmacological inhibition of CCR2 blocks the trans-differentiation induced by CCL2 but not CYTL1. Therefore, future work is needed to identify the CYTL1 receptor(s) and their function in the regulation of bone mass.

## Materials and methods

### Mice, μCT, bone histomorphometry, and ovariectomy

The utilized C57BL/6 *Cytl1*^*−/−*^ mice were as described previously^8^. The animals were housed in a pathogen-free facility at the Gwangju Institute of Science and Technology (GIST), and all experiments were approved by the GIST Animal Care and Use Committee. WT and *Cytl1*^−*/*−^ male mice (5-week-old or 5-month-old) were subjected to bone analyses. For μCT and bone histomorphometric analysis, femurs were scanned using a μCT scanner (Skyscan 1172) at 50 kV and 200 μA with a 0.5-mm aluminum filter and a detection pixel size of 10.7 μm. Images were captured every 0.7° through a 180° rotation of the bone. The trabecular bone volume, thickness, separation, and number were quantified using the 3-dimensional CT analyzer software (CTAN; Skyscan). For bone histomorphometric analysis, femurs were fixed in 4% paraformaldehyde and decalcified with 0.5 M EDTA (pH 7.4). The samples were dehydrated in a graded ethanol series and embedded in paraffin. Five-μm-thick sagittal sections were cut and subjected to H&E or TRAP staining^38^. For the quantification of N.Ob./B.Pm and N.Oc./B.Pm, three slides were selected for each mouse and the numbers of osteoblasts and TRAP-positive cells were each counted from 10 random microscopic fields per slide^39^. Ten-week-old female WT and *Cytl1*^*−/−*^ mice were subjected to ovariectomy (OVX)^40^, with sham-operation used as a control. Dorsal midline incisions were made under sterile conditions, and both ovaries were removed. μCT and bone histomorphometric analyses were performed at 8 weeks after OVX.

### Calcein double labeling

Mice were intraperitoneally (IP) injected with 5 mg/kg of body weight of calcein at 11 and 2 days before killing. The femurs were fixed for 1 day, incubated in 5% potassium hydroxide for 4 days, dehydrated, embedded, and sliced at a thickness of 6 μm. The distance between the parallel calcein signals was measured to yield the MAR (mineral apposition rate, μM/day). The single-labeled bone surface (sLS), double-labeled bone surface (dLS), and total bone surface (BS) were measured separately. The MS/BS (mineral apposition rate per bone surface) was obtained using the equation: (dLS + sLS/2)/BS. The BFR (bone formation rate) was calculated by multiplying MAR and MS/BS.

### Osteogenic, adipogenic, and chondrogenic differentiation of hMSCs

hMSCs were purchased from Lonza and cultured in ɑ-MEM supplemented with 10% fetal bovine serum and 1% penicillin/streptomycin. Passage 5 and 6 hMSCs were used for osteogenic, adipogenic, and chondrogenic differentiation. Osteogenesis was induced for up to 21 days in ɑ-MEM supplemented with 50 μM ascorbic acid, 10 mM β-glycerophosphate, and 0.1 μM dexamethasone. Osteogenesis was determined by detecting Alizarin red S staining, measuring ALPL activity, and analyzing the osteogenic markers, ALPL, IBSP, OCN, OPN, TAZ, and RUNX2. To quantify mineralization, Alizarin red S-stained cells were incubated with 10% cetylpyridinium chloride (to release calcium-bound Alizarin red S), and absorbance was measured at 550 nm and normalized with respect to the protein content. Relative mineralization was calculated by dividing the Ad-CYTL1 value by the Ad-C value. ALPL activity was measured with an alkaline phosphatase colorimetric assay kit (Abcam). Relative ALPL activity was obtained by dividing the ALPL activity of the indicated culture day with that obtained on day 3 in cells infected with Ad-C. Adipogenesis was induced by adding 0.5 mM 3-isobutyl-1-methylxanthine, 1 μM dexamethasone, and 200 μM indomethacin to the ɑ-MEM. Adipogenesis was determined by Oil red O staining and detection of the adipogenic markers, FABP4, PPARγ, and CEBPα. Chondrogenesis was induced by performing pellet culture of hMSCs in ɑ-MEM supplemented with 10 ng/ml TGF-β3, 50 ng/ml BMP-2, 1.25 mg/ml BSA, 1% insulin-transferrin-selenium (ITS), 1 mM sodium pyruvate, 50 μM ascorbic acid, 50 μM l-proline, and 0.1 μM dexamethasone. Chondrogenic differentiation was determined by Alcian blue staining and detection of the chondrogenic markers, SOX9, type II collagen (collagen-II, COL2A1), and aggrecan^8^.

### Osteoclastogenesis

Osteoclastogenesis of BMMs was induced as described previously^38^. BMMs were isolated from the femurs and tibias of 8-week-old WT and *Cytl1*^*−/−*^ mice. The cells were cultured in α-MEM for 1 day, and non-adherent cells were collected and incubated for an additional 3 days with 30 ng/ml M-CSF. Cells at this stage were considered to be M-CSF-dependent bone marrow macrophages (BMMs) and were used as osteoclast precursors, or were re-seeded in 24-well plates and induced to differentiate into osteoclasts by 5 days of treatment with 30 ng/ml recombinant M-CSF and 100 ng/ml RANKL (both from PeproTech). Osteoclasts were identified by staining for TRAP activity using a Leukocyte Acid Phosphatase Assay Kit (Sigma) according to the manufacturer’s instructions. The numbers and sizes of TRAP-positive cells were determined for 10 different random microscopic fields in each well.

### Cell proliferation and apoptosis

Cell proliferation was measured using a Cell Proliferation ELISA, BrdU (colorimetric) Kit (Roche). To assess the proliferation of undifferentiated hMSCs, cells were seeded in triplicate to a 96-well plate and treated with reCYTL1, infected for 2 h with 400 MOI of Ad-C or Ad-CYTL1, or infected for 12 h with 25 MOI of lentivirus containing control shRNA or shRNA specific for human CYTL1. The cells were incubated for an additional 24, 36, and 48 h. AKT inhibitors were added 30 min before reCYTL1 treatment or the various infections, and BrdU was incorporated during the last 4 h of culture. The culture medium was removed and the cells were fixed and incubated for 90 min with the provided anti-BrdU-POD. The cells were washed, the substrate was added, and the reaction product was measured at 370 nm with a reference wavelength of 492 nm. To assess cell viability during osteogenesis, we used an MTS-based Cell Titer 96 Aqueous One Solution Cell proliferation assay kit (Promega). Undifferentiated hMSCs were infected with 400 MOI of Ad-C or Ad-CYTL1 and allowed to undergo osteogenic differentiation for 3, 7, and 14 days. The MTS solution was added, cells were harvested 4 h later, and the absorbance was measured at 490 nm. Apoptotic cell death was detected by TUNEL staining using an in situ cell death detection kit (Roche). TUNEL-positive cells were counted from 10 random microscopic fields.

### Adenovirus, lentivirus, shRNA, siRNA, and reCYTL1

The adenovirus bearing human CYTL1 (Ad-CYTL1) was purchased from Vector Biolabs. Empty virus was used as a control (Ad-C). hMSCs were infected with the relevant adenovirus at an MOI of 400 for 2 h and then incubated for an additional 36 h or 14 days, as indicated. Two different lentiviruses bearing shRNAs (shRNA1 and shRNA2) were purchased from Sigma. The sequences of these shRNAs and the control (non-mammalian) shRNA are presented in Table S1. hMSCs were infected with 25 MOI of lentivirus, and infected cells were selected by incubation with puromycin (4 μg/ml) for 4 days. The level of CYTL1 in hMSCs was also down-regulated by transfection of a mixture of three different siRNAs specific for human CYTL1, as presented in Supplementary Table S1. reCYTL1 (Abcam) was added to the osteogenic medium, and the medium was replaced every 3 days.

### Reverse transcription-polymerase chain reaction, quantitative RT-PCR, western blotting, and immunofluorescence

Total RNA was isolated using the TRI reagent (Molecular Research Center) and reverse transcribed. The generated cDNA was PCR amplified with Taq polymerase (iNtRON) and the target mRNAs were quantified by quantitative RT-PCR (qRT-PCR) using a CFX Connect (Bio-Rad) and SYBR Premix Ex Taq (Takara Bio). The utilized PCR primers and conditions are presented in Supplementary Table S2. For Western blotting, total cell lysates were obtained with RIPA buffer and three freeze-thaw cycles. Western blot analyses were performed to detect the indicated proteins. The utilized antibodies were purchased from BD Bioscience (anti-β-catenin, anti-ERK, and anti-GSK3β), Abcam (anti-β-actin), and Cell Signaling (anti-AKT, anti-pAKT, anti-p38, anti-pp38, anti-pERK, anti-pGSK3β, anti-GAPDH, anti-BCL2, anti-BAX, anti-RUNX2, anti-p53, and anti-TAZ).

### Reporter gene assay

A mouse osteocalcin II (OG2) promoter-luciferase construct (pOG2-Luc) was used to measure RUNX2 transcriptional activity^41^. pCMV-*Runx2* and pcDNA3.1-*Cytl1* were as previously described^8^. To analyze the RUNX2‐dependent trans-activity of pOG2-Luc, we plated hMSCs to 6-well plates and performed transfections on days 0, 3, and 7 of osteogenesis using Lipofectamine 2000 (Invitrogen). The transfected cells were incubated for 36 h, and luciferase activity was determined with a Promega Luciferase Assay system.

### Fluorescence activated cell sorting (FACS) analysis

To identify the immune phenotypes of hMSCs subjected to CYTL1 overexpression or knockdown, FACS was performed. hMSCs were infected with 400 MOI of Ad-C or Ad-CYTL1 for 2 h and cultured for an additional 48 h. Alternatively, cells were infected with 25 MOI of control lentivirus shRNA or the two different *CYTL1* shRNAs. shRNA-infected hMSCs were selected by puromycin. hMSCs infected with Ad-CYTL1 or the lentivirus-based CYTL1 shRNA were suspended in PBS and incubated for 30 min with anti-CD44, anti-CD29, or anti-CD90 (Abcam). The cells were washed and incubated with goat anti-mouse Alexa Fluor 488 or goat anti-rabbit Alexa Fluor 594 (Invitrogen). The cells were washed and immediately examined using a FACS Canto II flow cytometer (BD Biosciences). The data were analyzed with the FlowJo software (Tree Star).

### Statistical analysis

All experiments were repeated independently at least three times. For the in vitro studies, each independent experiment was conducted using hMSCs derived from different donors and mouse bone marrow cells derived from an individual mouse. For qRT-PCR data expressed as relative fold changes, the two-tailed Student’s *t*-test and analysis of variance (ANOVA) with *post-hoc* tests were used for pair-wise comparisons and multi-comparisons, respectively. The results are presented as means ± SEM, and probability values below 0.05 were considered statistically significant (*P* < 0.05). For the in vivo experiments, each independent trial was conducted using a pair of WT and *Cytl1*^*−/*−^ mice. The Student’s *t*-test was used to compare bone parameters.

## Supplementary information

Supplemental materials
