# Supplementary material for: CYTL1 regulates bone homeostasis in mice by modulating osteogenesis of mesenchymal stem cells and osteoclastogenesis of bone marrow-derived macrophages
Source: Cell Death Dis. 2019 Jan 18;10(2):47. doi: 10.1038/s41419-018-1284-4 (PMC6362050; doi:10.1038/s41419-018-1284-4)
Supplement: Supplementary file 1 — Supplemental materials [file 41419_2018_1284_MOESM1_ESM.pdf]

Supplementary Information

CYTL1 regulates bone homeostasis in mice by modulating osteogenesis of mesenchymal stem cells and osteoclastogenesis of bone marrow-derived macrophages

Youngnim Shin[1], Yoonkyung Won[1], Jeong-In Yang[1], Jang-Soo Chun[1]

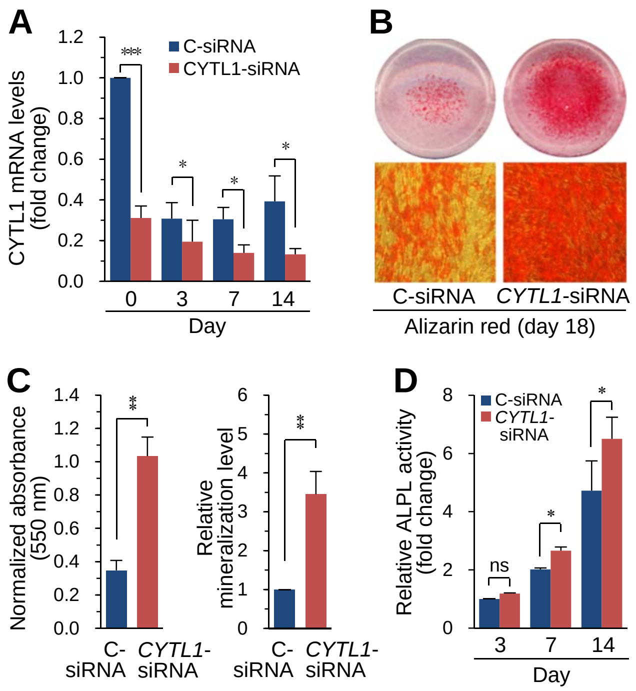

Fig. S1 Knockdown of CYTL1 promotes the osteogenesis of hMSCs. hMSCs were transfected with 100 nM of control or CYTL1-targeting siRNAs and cultured under osteogenic-differentiating conditions for the indicated number of days (**a**, **d**) or for 18 days (**b**, **c**). **a** CYTL1 mRNA levels were quantified by qRT-PCR analysis (n = 8). **b, c** Representative images of alizarin red S staining (**b**, n = 6). The absorbance of the released alizarin red S was normalized with respect to the protein content (left panel), and the relative value was obtained by dividing the value of Ad-*CYTL1* by that of Ad-C (right panel) (**c**, n = 6). **d** Relative ALPL activity (n = 5). Data represent the means ± SEM of the indicated number of independent experiments; *p <0.05, **p<0.005, ***p<0.0005 determined by two-tailed t-test. ns, not significant.

Table S1. Sequences of the utilized shRNA and siRNA

RNA	Species	Sequence (5´-3´)
CYTL1 shRNA-1	Human	CCGGGTACCTGGACATACACAATTACTCGAGTAATTGTGTAT GTCCAGGTACTTTTTG
CYTL1 shRNA-2	Human	CCGGGTAGATTCCTTGAAGGACAAACTCGAGTTTGTCCTTC AAGGAATCTACTTTTTG
CYTL1 siRNA-1	Human	Sense: GGUUUAAUGGAGUAAUGGU<br>Antisense: ACCAUUACUCCAUUAAACC
CYTL1 siRNA-2	Human	Sense: GUUAGAUACACAGCAUGUU<br>Antisense: AACAUGCUGUGUAUCUAAC
CYTL1 siRNA-3	Human	Sense: ACACCAUCAUGAACUCGUU<br>Antisense: AACGAGUUCAUGAUGGUGU
BAX siRNA-1	Human	Sense: GAGUGGCAGCUGACAUGUUUUCUGA<br>Antisense: GGCUCACCGUCGACUGUACAAAAGACU
BAX siRNA-2	Human	Sense: GUGGGCAUUUUUCUUACUUUUGUAA<br>Antisense: UGCACCCGUAAAAAGAAUGAAAACAUU

Table S2. PCR primers and conditions

Gene Symbol	Species	Strand	Sequence (5´-3´)	At (°C)	Size (bp)
ACAN	Human	Sense<br>Antisense	GCCTTGAGCAGTTCACCTTC<br>CTCTTCTACGGGGACAGCAG	60	395
ALPL	Human	Sense<br>Antisense	AGACTGCGCCTGGTAGTTGT<br>CCACGTCTTCACATTTGGTG	58	196
BAX	Human	Sense<br>Antisense	AACTGGTGCTCAAGGCCCTGTG<br>GCCTCAGCCCATCTTCTTCCAG	63	276
CEBPA	Human	Sense<br>Antisense	GCAAGGCCAAGAAGTCGGTGGAC<br>TGCCCATGGCCTTGACCAAGGAG	58	252
COL2A1	Human	Sense<br>Antisense	CAGTTGGGAGTAATGCAAG<br>GCCTGGATAACCTCTGTG	58	300
CYTL1	Human	Sense<br>Antisense	AGATCACCCGCGACTTCAAC<br>TTAGCGCTGACGATCTGGC	60	302
FABP4	Human	Sense<br>Antisense	TATGAAAGAAGTAGGAGTGG<br>ACCACCAGTTTATCATCCTC	58	288
GAPDH	Human	Sense<br>Antisense	CGTCTTCACCACCATGGAGA<br>CGGCCATCACGCCACAGTTT	62	300
IBSP	Human	Sense<br>Antisense	AGTGAGAGGGCAGAGGAA ATAC<br>CCTCCTCCTCTTCTGAACTGTC	57	331
OCN	Human	Sense<br>Antisense	GAGGGT ATA AACAGTGCTGGAG<br>CCCAGCCATTGATACAGG TAG	53	244
OPN	Human	Sense<br>Antisense	TTGCAGTGATTTGCTTTTGC<br>ACACTATCACCTCGGCCATC	60	439
PPARG	Human	Sense<br>Antisense	AGACAACAGACAAATCACCAT<br>CTTCACAGCAAACTCAAACTT	58	401
RUNX2	Human	Sense<br>Antisense	CTCACTACCACACCTACCTG<br>TCAATATGGTCGCCAAACAGATTC	58	270
SOX9	Human	Sense<br>Antisense	GGCAGCTGTGAACTGGCCA<br>GCACACGGGGAACTTGTC	62	408
TAZ	Human	Sense<br>Antisense	AGTACATGAACCACCTGACCG<br>TGTGCCTGCCTGTGTCTAGAAC	60	321
Acp5	Mouse	Sense<br>Antisense	CACGATGCCAGCGACAAGAGGTTC<br>AAACGTAGTCCTCCTTGGCTGCTGC	58	366
Gapdh	Mouse	Sense<br>Antisense	TCACTGCCACCCAGAAGAC<br>TGTAGGCCATGAGGTCCAC	60	450
Nfatc1	Mouse	Sense<br>Antisense	ACCACTCCACCCACTTCTGACTTC<br>AGCTGTAGCGTGAGAGGTTCATTC	58	349

Ocstamp	Mouse	Sense<br>Antisense	CTCTCTCTGTGGTCTCTTCGTCTTC<br>TGTGAAGGCGGAAGGCTGAG	60	343

S, sense; As, antisense; At, annealing temperature
